# Long-term efficacy and safety of bone cement-augmented pedicle screw fixation for stage III Kümmell disease

**DOI:** 10.1038/s41598-021-93013-1

**Published:** 2021-07-01

**Authors:** Guo-ye Mo, Teng-peng Zhou, Hui-zhi Guo, Yong-xian Li, Yong-chao Tang, Dan-qing Guo, Pei-jie Luo, Dong-xiao Li, Kai Yuan, Ling Mo, Shun-cong Zhang

**Affiliations:** 1 The First Affiliated Hospital of Guangzhou University of Chinese Medicine, Guangzhou, 510405 China; 2 Guangdong Second Traditional Chinese Medicine Hospital, 60 Hengfu Road, Yuexiu District, Guangzhou, 510000 Guangdong China; 3 The First Institute of Clinical Medicine, Guangzhou University of Chinese Medicine, Guangzhou, 510405 China

**Keywords:** Outcomes research, Orthopaedics

## Abstract

This study aimed to evaluate the efficacy and safety of bone cement-augmented pedicle screw fixation for stage III Kümmell disease. Twenty-five patients with stage III Kümmell disease who received bone cement-augmented pedicle screw fixation at the First Affiliated Hospital of Guangzhou University of Chinese Medicine between June 2009 and December 2015 were enrolled. All patients were females with a history of osteoporosis. The vertebral Cobb angle (V-Cobb angle), the fixed segment Cobb Angle (S-Cobb angle), pelvic parameters, visual Analogue Scale (VAS) score, and Oswestry Disability Index (ODI) were assessed preoperatively, postoperatively and at the final follow-up. Complications, loosening rate, operation time, and intraoperative bleeding were recorded. The average lumbar vertebral density T-value was − 3.68 ± 0.71 SD, and the average age was 71.84 ± 5.39. The V-Cobb angle, S-Cobb angle, and Sagittal Vertical Axis (SVA) were significantly smaller postoperatively compared to the preoperative values. The VAS and ODI at 1 month after surgery were 3.60 ± 1.00 and 36.04 ± 6.12%, respectively, which were both significantly lower than before surgery (VAS: 8.56 ± 1.04, ODI: 77.80 ± 6.57%). Bone cement-augmented pedicle screw fixation is a safe and effective treatment for stage III Kümmell disease. It can effectively correct kyphosis, restore and maintain sagittal balance, and maintain spinal stability.

## Introduction

The frequency of Kümmell disease, a complication of osteoporotic vertebral fracture (OVF), is increasing globally^1^. Since the condition was first described by Herman Kümmell in 1891, the literature has referred to Kümmell disease as being characterized by delayed osteonecrosis after trauma^2^, nonunion of compression fracture^3^, delayed collapse of OVF^4^, intravertebral pseudarthrosis^5^, and vertebral vacuum sign^6^. A history of minor trauma was also present in some patients. However, patients without a history of trauma, gradually develop activity-related back pain or lower extremity nerve symptoms after a few weeks or months of an asymptomatic period.

The development of Kümmell disease involves the following 3 stages: Stage I, spinal vertebral bodies are slightly injured due to minor trauma or in the absence of trauma; Stage II, vertebral bodies experience dynamic instability in the cleft area, followed by fracture and bone collapse; and Stage III, the compressed fractured vertebral body compresses the posterior spinal cord, leading to continuous back pain and other neurological symptoms^7^.

The current treatment strategies for Kümmell disease remain controversial because there are no specific treatment criteria. Various surgical strategies have been developed, including percutaneous kyphoplasty (PKP) or vertebroplasty (PVP), anterior-only, posterior-only, and combined anterior and posterior procedures^8–10^. For stages I and II Kümmell disease, adequate pain relief, vertebral body height restoration, and kyphosis correction are achieved with PKP and PVP^11,12^. However, stage III Kümmell disease patients with spinal canal stenosis, particularly with nerve damage, PKP or PVP are less effective, with a higher risk of cement leakage, and potential severe neurological damage. The final aim of treatment for patients with mild spinal canal stenosis is to relieve back pain, prevent further collapse of the affected vertebrae, and delay neurological deficits^13^.

However, for osteoporotic vertebrae, pedicle screw loosening and internal fixation failure are common postoperative complications^14^. Varieties of internal fixation methods such as the use of expansion screws and the increase of fixed segments have been proposed to reduce the risk of failure of common pedicle screw fixation in osteoporosis patients^14^. Bone cement-augmented pedicle screw fixation have been paid more and more attention by scholars in recent years because of its good pull-out resistance and fixation. Biomechanical tests have proved that bone cement reinforced pedicle screws can improve the pull-out resistance of 147–278%^15–17^. At present, there is no standard surgical option or single effective treatment. We aimed to address this gap by retrospectively analyzing groups of patients in our hospital to investigate the efficacy of bone cement-augmented pedicle screw fixation for stage III Kümmell disease.

## Materials and methods

**Figure 1:**
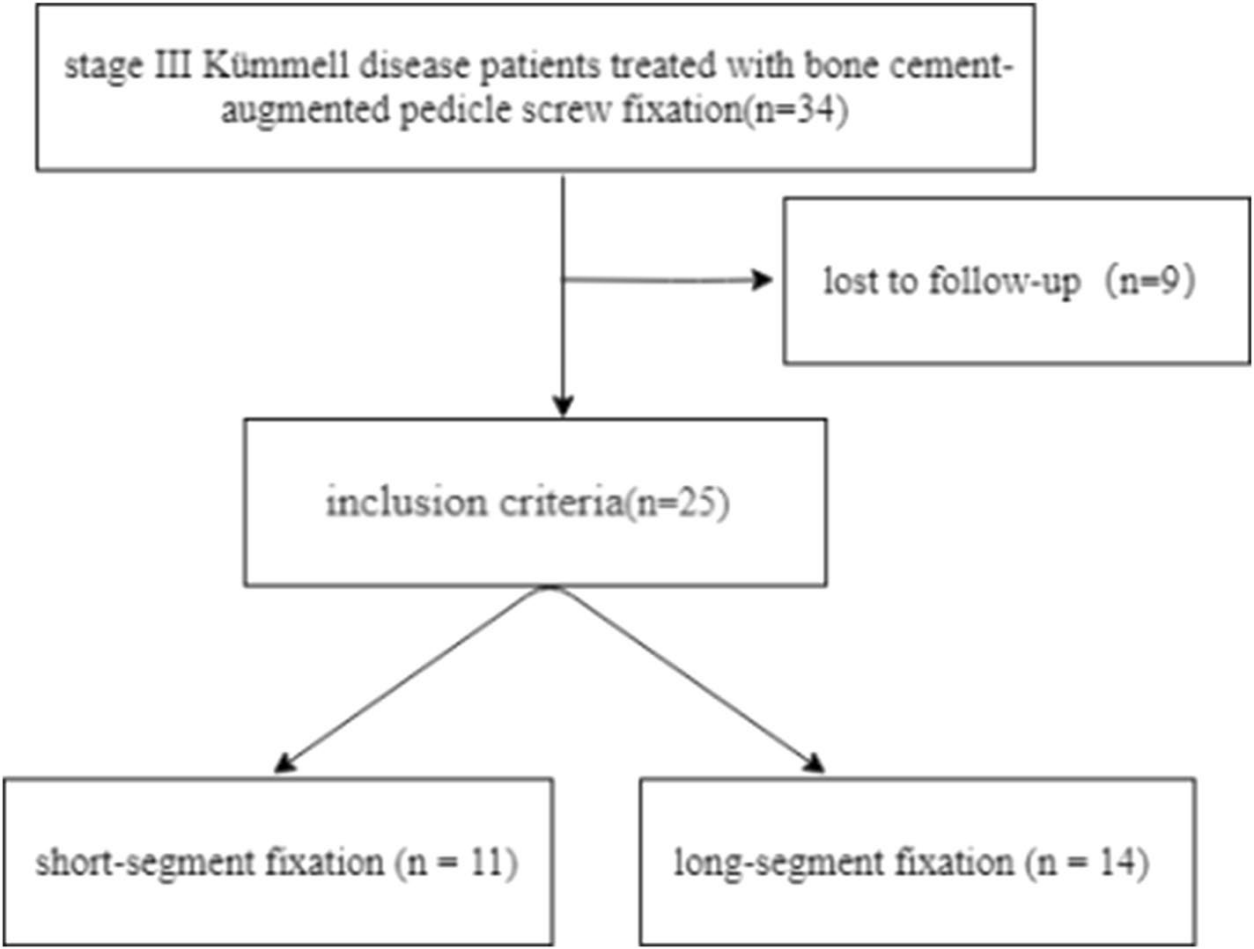
The research methodology flow chart.

This study was approved by the Ethics Committee of The First Affiliated Hospital of Guangzhou University of Chinese Medicine.The IRB number is NO. ZYYECK (2017)041. Written informed consent was obtained for each participant. From June 2009 to December 2015, patients with stage III Kümmell disease who visited in our hospital for bone cement-augmented pedicle screws were recruited. The inclusion criteria were: (1) diagnosis with the stage III Kümmell disease by symptoms, signs, and imaging examinations; (2) lumbar vertebral bone mineral density (BMD) of T ≤  − 2.5 SD measured by dual energy X-ray absorptiometry; (3) with or without neurological symptoms. Exclusion criteria included: (1) pathological vertebral fractures such as tumors or infections, and (2) serious medical diseases or intolerance to operation. The research methodology fow chart is shown in Fig. 1.

### Operative methods

Through the posterior median approach to the spine, the intersection of the midpoint of the transverse process of the vertebral body and the vertical line of the outer margin of the superior articular process was exposed. Cannulated pedicle screws were inserted into the vertebrae under the monitoring of a C-arm X-ray machine, and then 1.5–2.5 ml of bone cement was injected into each screw channel. The cannulated pedicle screws are hollow and they have some lateral holes in the tip of the screw, thus the bone cement can fill up the cancellous bone around the screws. These screws come from Shanghai Reach Medical Instrument Co. Ltd. The specification of pedicle screw was determined preoperatively by measuring the diameter of the pedicle.

### Postoperative management

At early stage after operation, the patients were required to perform both lower limbs exercises on the bed. The drainage tube was removed when the amount of drainage was less than 50 ml/day. All patients began to get out of bed and walk with the protection of waist circumference 3 or 4 days after the operation. Patients were required to wear a waist circumference within first month after operation. Cephalosporin was used once intraoperatively to prevent infection, and antibiotics were not routinely used postoperatively. After operation, standard anti-osteoporosis treatment (calcium, vitamins D and diphosphate).

### Data collection and radiographic assessment

#### Evaluation of clinical efficacy

The VAS and ODI scores were recorded by ward clinicians during outpatient visits or telephone follow-up. The operation time and blood loss were obtained from medical records. The incidence of complications, such as nerve injury, dural tearing, and bone cement leakage were also recorded.

#### Imaging evaluation

The V-Cobb angle and S-Cobb angle of the injured vertebrae were measured by lateral X-ray and compared at the preoperative, postoperative and final follow-up appointments. Loosening of the pedicle screw was observed on CT or X-ray film during the final follow-up. All spinopelvic parameters were measured twice on total spine photography by two orthopedists, independently, and the mean values were used for analysis. The degree of lumbar lordosis (LL) was assessed from the inferior endplate of T12 to the superior endplate of S1^18^. Pelvic tilt (PT) was defined as the angle subtended by a line drawn from the midpoint of the sacral endplate to the center of the bicoxofemoral axis and a vertical plumb line extending from the bicoxofemoral axis^19^. Sacral slope (SS) was defined as the angle between the end plate of S1 and the horizontal line^19^. Pelvic incidence (PI) was defined as the angle between the line perpendicular to the sacral endplate at its midpoint and the line connecting this point to the midpoint of femoral heads axis^19^. The sagittal vertical axis (SVA) was measured by the horizontal distance from the S1 postero-superior corner to the C7 PL^18^^.^

### Statistical analysis

IBM SPSS 19.0 Software was used to analyze the data. All data are expressed as mean ± SD. One-way ANOVA followed by the Student–Newman–Keuls post hoc test was used, and p < 0.05 was statistically significant.

## Results

**Figure 2:**
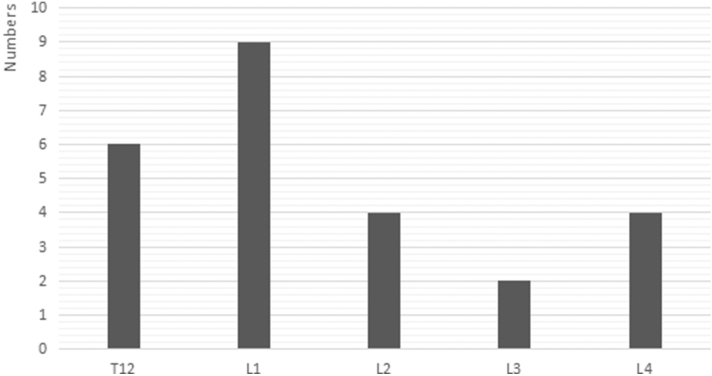
Distribution of the injured vertebrae.

**Figure 3:**
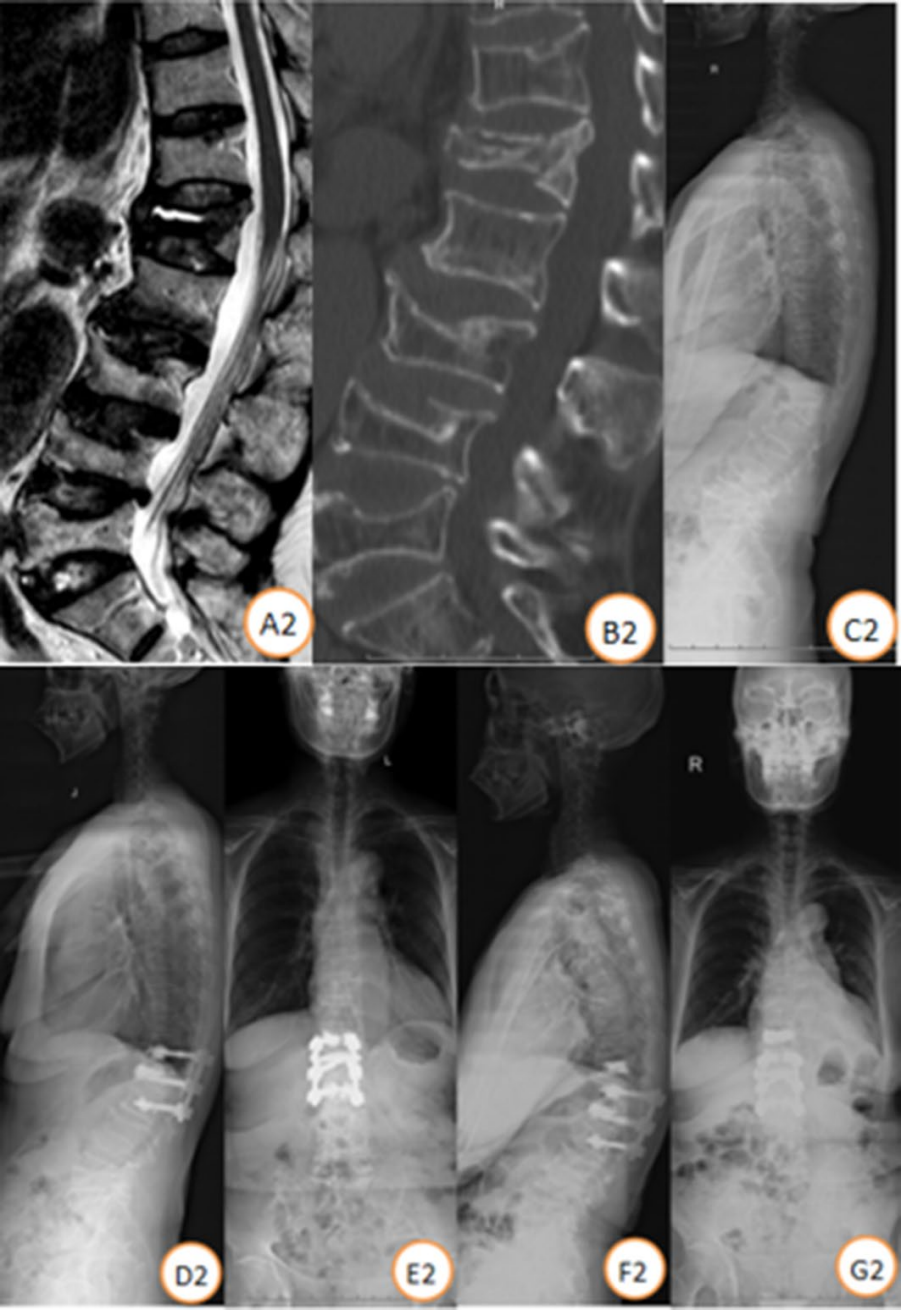
Case illustration of a patient. Female, 62 years, BMD (T score) was − 3.8SD. (**A2–C2**) Preoperative lateral X-ray and CT scan showed L1 vertebral fracture with, MRI showed low signal of the necrotic area on T2-weighted images. Follow-up standing X-ray at 3 months (**D2,E2**) and 4 year (**F2,G2**) post T12-L2 bone cement-augmented pedicle screw fixation.

**Figure 4:**
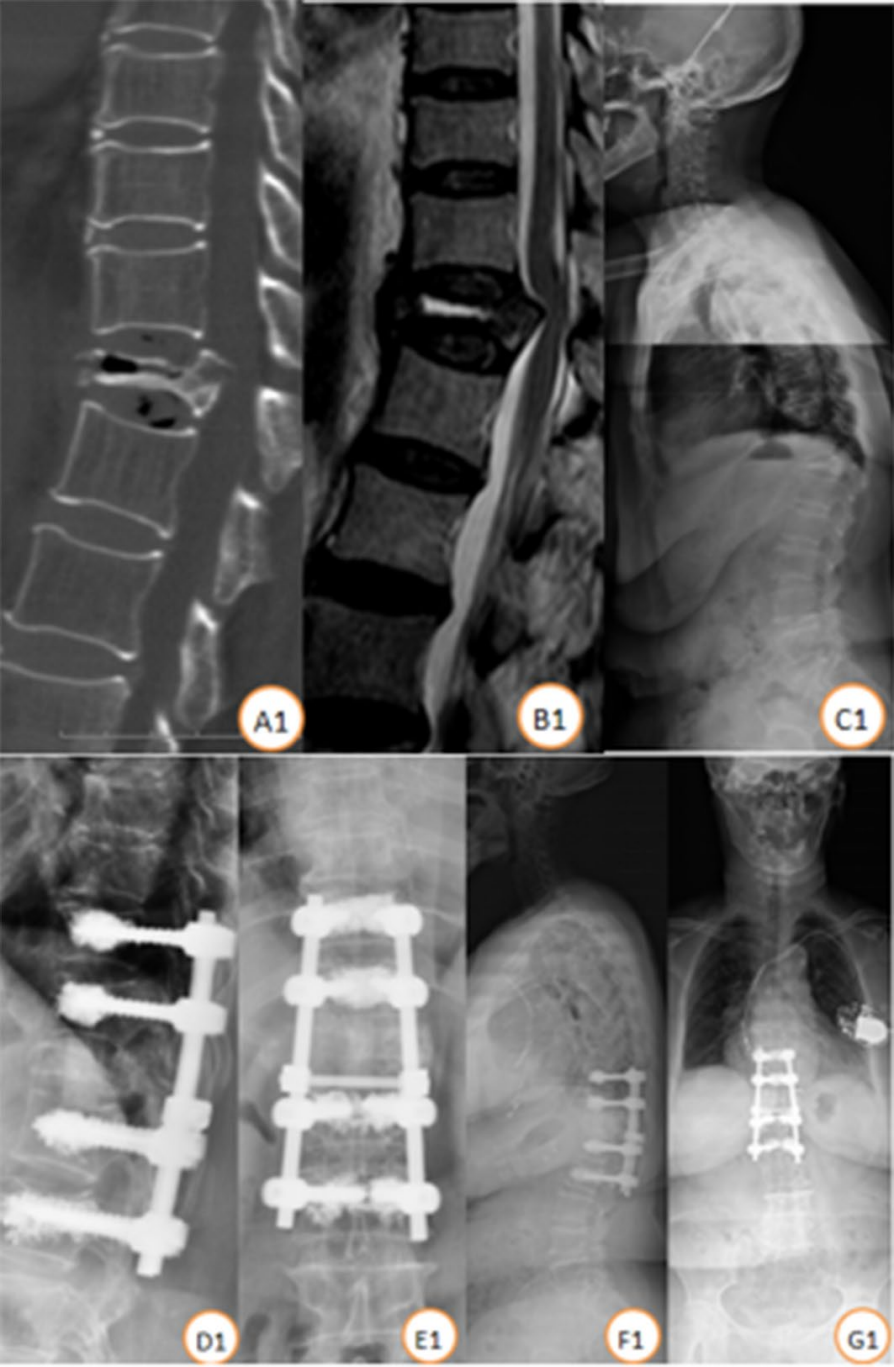
Case illustration of a patient. Female, 80 years, BMD (T score) was − 3.4SD. (**A1–C1**) Preoperative lateral X-ray and CT scan showed T12 vertebral fracture with, MRI showed low signal of the necrotic area on T2-weighted images. Follow-up standing X-ray at 2 year (**D1,E1**) and 5 year (**F1,G1**) post T10-L2 bone cement-augmented pedicle screw fixation.

There were 34 stage III Kümmell disease patients treated with bone cement-augmented pedicle screw fixation. A total of 25 patients were enrolled in this study (Fig. 2). Four patients died of other diseases, and 9 were lost to follow-up. The follow-up rate was 73.52%. All patients were females with a mean patient age of 71.84 ± 5.39 years and T scores of − 3.68 ± 0.71 SD. Patients were treated with short-segment fixation (n = 11) (Fig. 3) or long-segment fixation (n = 14) (Fig. 4) combined with posterolateral fusion. All methods were carried out in accordance with relevant guidelines and regulations in the manuscript.

**Figure 5:**
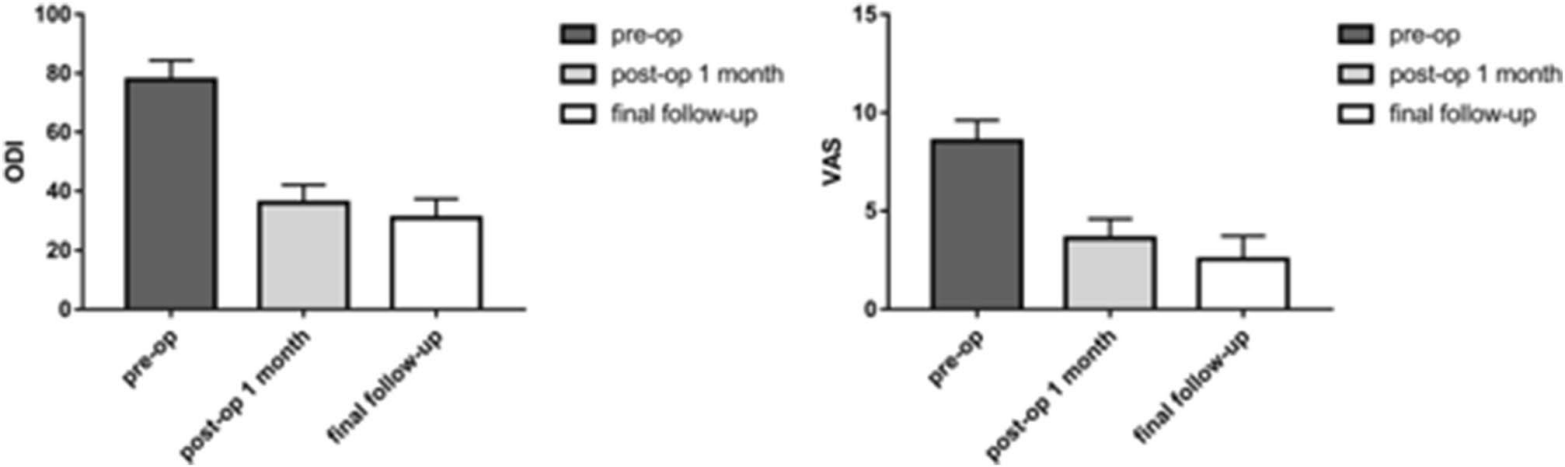
ODI and VAS scores. *ODI* Oswestry Disability Index, *VAS* visual analogue scale.

All patients underwent spinal surgery successfully. Two patients underwent a second surgery on day 6 and 7 after the first surgery because of poor efficacy, and were then discharged successfully. The surgical wound of three patients did not heal well, but they were discharged successfully after extended antibiotic treatment. During the operation, none of the patients experienced nerve injury or dural sac tearing. A total of 178 pedicle screws were used, and all were reinforced with bone cement. Among them, 45 screws showed bone cement leakage beside the vertebra or along the vertebral body vein, with a leakage rate of 25.28%. No patients had serious complications such as nerve compression or pulmonary embolism due to bone cement leakage. During the follow-up, we found 7 fixed segments with adjacent vertebral fractures, however, these were relieved with vertebroplasty. No pedicle screw loosening was found during follow-up. Before surgery, most patients showed signs of nerve compression, but symptoms were significantly recovered after surgery (Table 1). Postoperative and follow-up ODI and VAS scores were significantly lower than preoperative scores (Fig. 5).

**Table 1:** Frankel classification.

	A	B	C	D	E
Preoperative	0	1	3	17	4
Postoperative	0	0	1	1	23

Details of the patients’ demographic data are summarized in Table 2. No significant differences were observed in PI, PT, LL, and SS (Table 3).The V-Cobb angle, S-Cobb angle, and SVA of patients at the post-operative and the final follow-up appointments were significantly lower than the pre-operative appointment, no significant difference was observed between the post-operative and final follow-up appointments (Table 4).

**Table 2:** Baseline patient data.

Cases	25
Sex (male/female)	0/25
Age (years)	71.84 ± 5.39
Bone mineral density	− 3.68 ± 0.71
Follow-up (months)	43.08 ± 26.51
Operation time (min)	261.44 ± 54.27
Blood loss (ml)	376.40 ± 227.54

**Table 3:** Radiographic results.

	*n*	Preop	Postop	Final follow-up	*F*	*P-*value
V-Cobb angle	25	25.60 ± 10.71	10.81 ± 6.41	15.65 ± 20.99	7.686	0.001
S-Cobb angle	25	25.77 ± 10.71	13.26 ± 10.67	15.57 ± 9.76	10.271	0.000
PI	25	48.62 ± 12.41	50.60 ± 13.47	49.41 ± 9.76	0.145	0.865
PT	25	18.76 ± 8.25	22.00 ± 12.63	19.73 ± 8.53	0.579	0.563
LL	25	32.08 ± 14.46	33.92 ± 10.54	34.06 ± 12.56	0.166	0.848
SS	25	31.38 ± 10.64	28.75 ± 4.04	29.38 ± 5.93	0.720	0.491
SVA(cm)	25	5.76 ± 2.14	3.40 ± 0.93	3.45 ± 1.67	13.863	0.000

*PI* pelvic incidence, *LL* lumbar lordosis, *PT* pelvic tilt, *SS* sacral slope, *Preop* preoperative, *postop* postoperative, *SVA* sagittal vertical axis.

**Table 4:** Comparison of radiographic results at different time points.

	Preop vs post-op		Preop vs final follow-up		Postop vs final follow-up	
	*T* value	*P* value	*T* value	*P* value	*T* value	*P* value
V-Cobb angle	3.867	0.000	2.495	0.015	1.372	0.174
S-Cobb angle	4.259	0.000	3.472	0.001	0.787	0.434
PI	0.536	0.594	0.215	0.831	0.321	0.749
PT	1.048	0.299	0.313	0.755	0.735	0.465
LL	0.480	0.633	0.516	0.608	0.036	0.972
SS	1.148	0.255	0.876	0.385	0.273	0.786
SVA	4.613	0.000	4.505	0.000	0.108	0.914

*PI* pelvic incidence, *LL* lumbar lordosis, *PT* pelvic tilt, *SS* sacral slope, *Preop* preoperative, *postop* postoperative, *SVA* sagittal vertical axis.

## Discussion

Kümmell disease was first described in 1891 as an uncommon complication of osteoporotic vertebral fracture, more frequently encountered in patients with severe osteoporosis that have taken long-term courses of corticosteroids or sustained a spinal injury^20^. Kümmell disease is different from fresh vertebral fracture, and intra-bone clefting is the most important characteristic used to diagnose Kümmell disease. Kümmell disease mostly occurs in women^13^. The thoracolumbar junction, particularly the T12 vertebra, is the most commonly affected vertebral segment^21^. In our study, 76% of injured vertebra were in the thoracolumbar segment. Thoracolumbar vertebral body has large range of motion and stress concentration, which easily leads to ischemic necrosis after osteoporotic vertebral fracture.

Different stages of Kümmell disease have unique pathological features. The early stages include avascular necrosis characterized by an accumulation of fluid and inflammatory exudate components^21^. During stage III, the compressed, fractured vertebral body compresses the posterior spinal cord, leading to continuous back pain and other neurological symptoms. Stage III Kümmell disease is characterized by the collapse of the posterior vertebral body wall, the formation of spinal canal stenosis, and dural sac compression^21^. Most stage III patients, including 84% of the patients in our study, have preoperative neurological symptoms. In our study, the posterior wall of the fractured vertebral body was fragmented and compressed into the dural sac in all patients.

The treatment of the Kümmell disease remains controversial. Most spinal surgeons suggest that Kümmell disease should be treated by operative interventions because conservative treatments are less effective and are associated with a high risk of complications and delayed neurological deficits^22^. For early stages patients without neurological symptoms, the aim of treatment is to preserve movement in the diseased vertebrae, and maintain the sagittal balance of the spine. PKP and PVP restore the height of the vertebral body and correct any deformities, which can help achieve satisfactory pain relief^23^. However, PVP and PKP are less suitable for stage III patients because the surrounding vertebral cortex has already been compromised, as well as a higher risk of severe nerve damage caused by bone cement leakage^13^. In our study, the bone cement leakage rate was 25.28%, but there were no serious complications, suggesting that short-term leakage is not damaging. Delayed cement displacement and further collapse have been reported in cases of Kümmell disease treated by cement augmentation alone, with poor bone incorporation of cement noted after a long-term follow-up^24,25^. Therefore, displacement of bone cement and further vertebral collapse may occur after PKP or PVP^26^. In our study, some patients underwent vertebroplasty for fractured vertebrae, but we did not find cement displacement during follow-up because bone cement-augmented pedicle screw fixation provided stability.

There are alternative treatment strategies for stage III Kümmell disease, but consensus regarding which is most feasible and effective is lacking. For stage III patients with severe stenosis of the spinal canal and neurological symptoms, the objective of surgery is to relieve cord compression, eliminate spinal instability, and restore the sagittal balance of the spine^12,27^. Many studies have suggested that the main factor contributing to delayed neurological deficits following vertebral collapse in the osteoporotic spine is instability at the fracture site, rather than mechanical compression of the spinal cord by bone fragments^28,29^. Therefore, maintaining spinal stability is important for treating stage III patients. Other studies suggested that modified posterior vertebral column resection surgery was an effective and safe surgical method to treat stage III Kümmell disease, especially for patients with kyphosis and obvious symptoms of nerve compression; however, the long-term clinical effects require additional evaluation. Anterior reconstruction and posterior osteotomy have also been proposed for the management of stage III Kümmell disease with neurological deficits^30^. Anterior reconstruction permits direct resection of bony fragments and provides anterior column support. Posterior osteotomy is a common treatment; the advantages include dissection of the posterior cortex by posterior spinal shortening osteotomy and correction of kyphosis^31,32^. Moreover, these major surgical interventions can be challenging in patients of advanced age, and confer numerous morbid complications and frequent instrumentation failure secondary to severe osteoporosis^4^. Traditional posterior long-segment fixation was not appropriate for stage III Kümmell disease because the procedure was associated with significant trauma and multiple complications, which are worrisome in elderly patients with comorbidities^33^. Compared with these methods, on the basis of laminar decompression to improve the neurological symptoms caused by spinal stenosis, and according to the patient’s fracture end reduction, using the bone cement-augmented pedicle screw fixation can further increase the stability and prevent the fracture from collapsing again. Lu et al.^34^ reported that the use of the bone cement-augmented pedicle screw fixation among the thoracolumbar fractures patients with satisfactory results. In our study, most patients showed signs of nerve compression preoperatively, but symptoms were significantly recovered after surgery. Postoperative and follow-up ODI and VAS scores were significantly lower than preoperative scores.

Leakage of bone cement is a common complication of bone cement-augmented pedicle screw fixation. Jassen et al.^35^ reported that among the 165 patients who underwent bone cement-augmented pedicle screw fixation, 110 (66.7%) had bone cement leakage without neurological symptoms, and 13 (7.9%) had pulmonary embolism, of which 5 cases (3%) had symptoms of pulmonary embolism requiring treatment, and 2 cases (1.2%) had symptoms of bone cement allergy. In our study, 45 of the 178 screws showed bone cement leakage beside the vertebra or along the vertebral body vein, with a leakage rate of 25.28%. In addition, although all the leakage of bone cement has no obvious clinical symptoms, there are some studies have shown that the leakage of bone cement in the intervertebral disc will increase the risk of secondary fractures of the adjacent vertebral body during the long term follow up^36,37^.

In order to avoid leakage of bone cement, we can take some measures as follows: (1) select a suitable screw. The data including the pedicle width, vertebral length and introversion angle should be measured before the operation; (2) reduce internal inclination of screw placement to avoid screw appearing in the middle of the vertebral body properly; (3) a proper time to inject the bone cement while the cement becomes at an appropriate timing; (4) X ray or/and CT would be used as monitoring during operation. By using small amount multiple injection method (0.1 ml cement injection per time) before another X-ray examination, we could make sure if the bone cement have leakage or not; (5) it is recommended that the amount of single nail bone cement should be about 1.5–2 ml to control the amount of bone cement injected and the number of reinforcement screws.

There is high risk of pedicle screw loosening because of the osteoporotic vertebra in stage III Kümmell disease. Considering the unsatisfactory performance of traditional pedicle screws in damaged spines, bone cement-augmented pedicle screw fixation strengthens the anti-pullout capability by injecting cement carefully through the screws into the vertebral body. The final follow-up results of SVA, V-Cobb angle and S-Cobb angle were significantly better than those preoperation. The sagittal balance of the spine and the local balance and stability of fractured vertebral body were corrected and maintained by increasing the stability of pedicle screw. In our study, there was no significant difference between postoperative and the final follow-up aboutresults of SVA, V-Cobb angle and S-Cobb angle, which means that bone cement-augmented pedicle screw fixation maintained the stability of the spine and pelvis of the stage III Kümmell disease patients. Posterior screw stress was markedly reduced because of the anterior support provided by the intravertebral cement, which can decrease the risk of internal fixation failure^18^.

There are some limitations to our study. First, it was a retrospective study at a single center with a small sample size. Thus, further studies with larger samples are needed to confirm our findings. Secondly, we observed long segmental fixation and short segmental fixation together; however, these two fixation methods may have different therapeutic effects.

## Conclusions

Bone cement-augmented pedicle screw fixation is a safe and effective treatment for stage III Kümmell disease. It effectively corrects kyphosis, and safely maintains the stability of the spine and sagittal balance. Further prospective and large sample clinical studies are needed to confirm them.
